# Radiation-Induced Osteocyte Senescence Alters Bone Marrow Mesenchymal Stem Cell Differentiation Potential via Paracrine Signaling

**DOI:** 10.3390/ijms22179323

**Published:** 2021-08-28

**Authors:** Linshan Xu, Yuyang Wang, Jianping Wang, Jianglong Zhai, Li Ren, Guoying Zhu

**Affiliations:** Department of Radiological Hygiene, Institute of Radiation Medicine, Fudan University, 2094 Xietu Road, Shanghai 200032, China; 19211140005@fudan.edu.cn (L.X.); 18211140008@fudan.edu.cn (Y.W.); jianpingwang@fudan.edu.cn (J.W.); jlzhai@fudan.edu.cn (J.Z.); 20211140001@fudan.edu.cn (L.R.)

**Keywords:** cellular senescence, senescence-associated secretory phenotype (SASP), irradiation, osteocytes, bone marrow mesenchymal stem cells (BMSCs)

## Abstract

Cellular senescence and its senescence-associated secretory phenotype (SASP) are widely regarded as promising therapeutic targets for aging-related diseases, such as osteoporosis. However, the expression pattern of cellular senescence and multiple SASP secretion remains unclear, thus leaving a large gap in the knowledge for a desirable intervention targeting cellular senescence. Therefore, there is a critical need to understand the molecular mechanism of SASP secretion in the bone microenvironment that can ameliorate aging-related degenerative pathologies including osteoporosis. In this study, osteocyte-like cells (MLO-Y4) were induced to cellular senescence by 2 Gy γ-rays; then, senescence phenotype changes and adverse effects of SASP on bone marrow mesenchymal stem cell (BMSC) differentiation potential were investigated. The results revealed that 2 Gy irradiation could hinder cell viability, shorten cell dendrites, and induce cellular senescence, as evidenced by the higher expression of senescence markers p16 and p21 and the elevated formation of senescence-associated heterochromatin foci (SAHF), which was accompanied by the enhanced secretion of SASP markers such as IL-1α, IL-6, MMP-3, IGFBP-6, resistin, and adiponectin. When 0.8 μM JAK1 inhibitors were added to block SASP secretion, the higher expression of SASP was blunted, but the inhibition in osteogenic and adipogenic differentiation potential of BMSCs co-cultured with irradiated MLO-Y4 cell conditioned medium (CM- 2 Gy) was alleviated. These results suggest that senescent osteocytes can perturb BMSCs’ differential potential via the paracrine signaling of SASP, which was also demonstrated by in vivo experiments. In conclusion, we identified the SASP factor partially responsible for the degenerative differentiation of BMSCs, which allowed us to hypothesize that senescent osteocytes and their SASPs may contribute to radiation-induced bone loss.

## 1. Introduction

With the rapid aging of the global population, research on effective treatment for aging-related diseases has been widely concerned with extending both life and health spans [1]. Advanced age is a natural physiological process that degenerates over time, and it is a key risk for most chronic diseases such as osteoporosis, which is a chronic disease associated with aging that can cause bone deterioration, bone atrophy, and refractory and non-healing fractures, as well as seriously reduce life quality and eventually lead to considerable mortality [2,3]. One of the main causes of age-related bone loss is an increase in senescent cells and their proinflammatory factors in the bone microenvironment, which may cause substantial pathogenic effects and ageing phenotypes [4,5].

In vivo studies have revealed that the accumulation of senescent cells in tissue can damage the self-repair of tissues, establish a senescent microenvironment, affect the physiological functions of surrounding normal cells, and lead to tissue dysfunction and diseases [6,7]. Cellular senescence, one of the most rapidly developing branches of science, was first proposed by Hayflick and described as irreversible cell cycle arrest due to continuous passage intended to prevent the aberrant proliferation of damaged cells [8], which is caused by telomere shortening and has been termed replicative senescence [9,10]. In contrast to original findings, more recent research has shown that similar phenotypes may be induced by the activation of a variety of stressors, such as oxidative stress and irradiation; this is termed stress cellular senescence. Radiotherapy has become an essential treatment for cancer patients, and there is growing evidence linking cancer radiotherapy to radiation-induced bone injury and cellular senescence [11,12]. Ionizing radiation induces DNA damage, chromosomal aberrations, increased amounts of reactive oxygen species, and cellular senescence in cells within the bone microenvironment, which leads to senescence-like conditions [13]. During the period of cancer treatment, with the occurrence of bone aging and tissue dysfunction, the adverse effects of senescence that cause dysfunction in other tissues appear to also occur in bone tissue; these include impaired osteoblast progenitor cell function, defective bone formation, and enhanced osteoclastogenesis [14].

Treatment with ionizing radiation or chemotherapeutic drugs may actuate therapy-induced senescence (TIS), which is caused by persistent DNA damage response and induces the expression of the cell cycle inhibitors *p16^INK4a^* and *p21^WAF1/CIP1^* [15]. Despite their growth stagnation and dysfunction, senescent cells remain metabolically active and continue to produce a variety of proinflammatory cytokines, chemokines, and extracellular matrix proteins that together create a toxic microenvironment termed the senescence-associated secretory phenotype (SASP) [16]. Furthermore, the SASP may contribute to additional senescent cell accumulation and further tissue dysfunction by spreading inflammatory factors to neighboring bystander cells. It has been shown that irradiation can induce cellular senescence and the apoptosis of cells in the bone microenvironment, as well as cause osteogenic dysfunction [17]. Meanwhile, available data suggest that the accumulation of senescent osteoblasts and osteocytes can be observed in bone loss caused by local radiotherapy, characterized by an increase in aging-associated β-galactosidase activity and telomere dysfunction-induced foci (TIF) [18]. Additionally, both bone marrow mesenchymal stem cells (BMSCs) and osteocytes are long-living cells, which are more susceptible to senescence upon stress and (as the most representative cells in bone tissue) release large amounts of SASP factors into the bone microenvironment as a result [19]. Furthermore, due to the multicellular regulation mechanism in bone tissue, senescent cells can perturb neighboring cells and spread the toxic SASP factors, both locally and systemically. However, the causal relationship between senescent cells and SASP secretion and the mechanisms that potentially alter bone remodeling in radiation-induced bone loss are not completely understood. In the present study, we focus on the radiation-induced senescence phenotype changes in osteocytes and their regulatory influence on the differentiation potential of BMSCs toward osteogenesis and adipogenesis, and we sought to identify the hallmarks and functions of the osteocyte SASP of cellular senescence within the bone microenvironment.

## 2. Results

### 2.1. Irradiation Impairs Dendritic Morphology and Cell Viability, and Induces Cell Apoptosis

**Figure 1:**
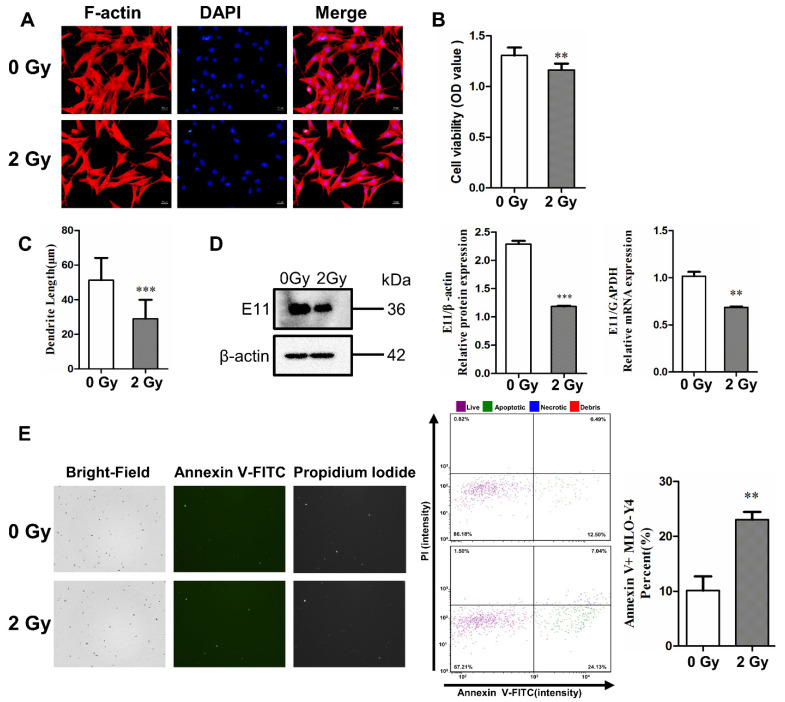
γ-ray irradiation inhibited dendrite elongation and cell activity, and it induced the apoptosis of osteocyte-like MLO-Y4 cells. (**A**) Morphological change in MLO-Y4 cells after 3 days post irradiation. F-actin (red) was stained with phalloidin, and nuclei (blue) were stained with DAPI; magnification = ×200. (**B**) Quantifying cell viability with Cell Counting Kit-8; *n* = 6. (**C**) The length change in dendritic structures in MLO-Y4 cells post irradiation; *n* = 5. (**D**) Detection of E11 mRNA and protein expression; *n* = 3. (**E**) Bright field (left column) and fluorescence images of Annexin V-FITC (middle column) and PI (right column). The images show an increase in the number of both apoptotic and necrotic cells post irradiation. Cytometer assay for the apoptosis of MLO-Y4 cells 3 days post irradiation; *n* = 3. PI: propidium iodide. Results are expressed as mean ± SD (** *p* < 0.01; *** *p* < 0.001 vs. 0 Gy).

Osteocytes have a unique stellate morphology and many dendritic protrusions that can expand to the bone surface, even to blood vessels. In this study, osteocyte-like MLO-Y4 cells were identified by F-actin fluorescence staining, and they were found to exhibit typical dendritic structures and E11 expression that represented early osteocytes (Figure 1A,D).

The F-actin fluorescence staining results show that most of the irradiated MLO-Y4 cells exhibited obvious morphological changes, including a polygonal shape and shortened dendrites compared to the control group. The dendrite length of MLO-Y4 was also quantified, and it was found to have shortened by 40.3% on average under 2 Gy irradiation, with a significant difference (*** *p* < 0.001; Figure 1C). To further investigate the inhibitory effect of radiation on dendrite elongation, we examined the mRNA and protein expression of E11, which is a dendrite marker in osteocytes. The level of E11 mRNA in MLO-Y4 cells was downregulated after irradiation (** *p* < 0.01; *** *p* < 0.001; Figure 1D). Meanwhile, cell viability was determined with a CCK-8 assay. The survivability of MLO-Y4 cells decreased 3 days post irradiation (** *p* < 0.01; Figure 1B), which proved the direct deterioration effect of irradiation on osteocytes.

Subsequently, the expression of Annexin V, a protein marker of apoptosis, was increased in irradiated MLO-Y4 cells (** *p* < 0.05; Figure 1E). Fluorescence intensity was plotted with Annexin V-FITC relative to PI, which revealed an increase in the number of both apoptotic and necrotic cells due to irradiation. The proportions of both necrotic cells (upper right quadrant) and apoptotic cells (lower right quadrant). This showed that the proportion of living cells decreased (86.18% vs. 67.21%), while apoptotic cells and necrotic cells increased (12.50% vs. 24.13% and 0.49% vs. 7.04%, respectively) after 2 Gy irradiation compared to the control group, as presented in Figure 1B. These results show that 2 Gy irradiation could increase the rate of apoptosis by 12.89% compared to the control group (Figure 1E), which demonstrates that irradiation induces the impairment of molecular biological functions in osteocytes in in vitro experiments.

### 2.2. Irradiation Induces Cellular Senescence and DNA Damage

**Figure 2:**
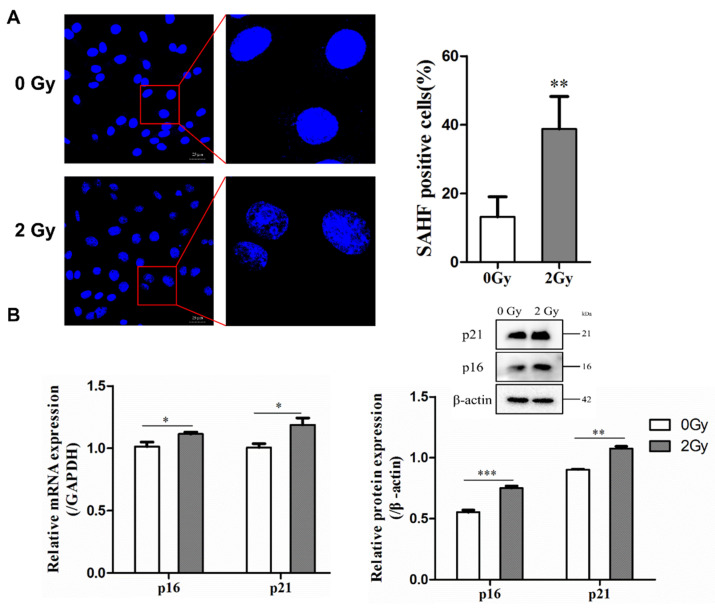

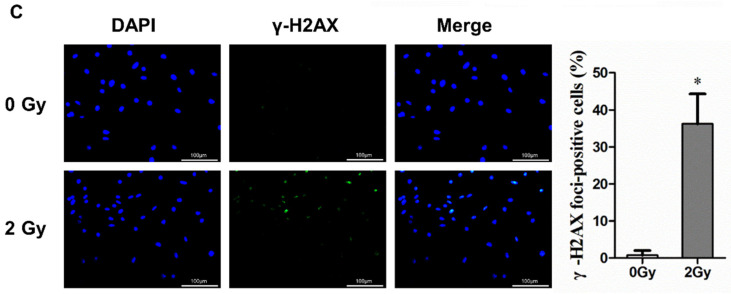
Radiation-induced cellular senescence and DNA damage in osteocyte-like MLO-Y4 cells. (**A**) Cells were stained with DAPI to observe SAHF formation; magnification = ×200. SAHF was examined in a total of four individual visual field images from each group. (**B**) Expression of p21 and p16 mRNA and proteins detected by RT-qPCR and Western blot analysis; *n* = 3. (**C**) Representative images of the γ-H2AX immunofluorescence staining (green) and nuclei (blue) of the irradiated MLO-Y4 cells; magnification = ×200. Percentage of γ-H2AX-foci-positive cells and the quantification of γ-H2AX foci in four separate visual field images for each group. Results are expressed as mean ± SD (* *p* < 0.05; ** *p* < 0.01; *** *p* < 0.001 vs. 0 Gy).

The destruction of chromatin structure in the nucleus, disruption of nuclear integrity, and formation of senescence-associated heterochromatic foci (SAHF) were observed by DAPI staining, thus confirming that radiation could induce structural changes in chromatin to form SAHF, which is a typic marker of senescent cells. According to the results, in the control group, the number of SAHF-positive cells accounted for 13.16%, while in the 2 Gy irradiation group, the number of SAHF-positive cells accounted for 38.79%, with a statistically significant difference (** *p*  <  0.01; Figure 2A). After irradiation-induced senescence in osteocytes, the protein and gene expression of p16 and p21 (which are senescence-related genes and proteins) were highly upregulated after 2 Gy irradiation in MLO-Y4 cells (* *p*  <  0.05; ** *p*  <  0.01; *** *p*  <  0.001; Figure 2B). Furthermore, it was found that irradiation could enhance the accumulation of γ-H2AX in osteocytes, a marker of DNA double-strand breaks. Compared to the control group, the percentage of γ-H2AX-positive cells was increased by 35.47% in the 2 Gy-irradiated group (* *p* < 0.05; Figure 2C).

### 2.3. Irradiation Enhanced SASP Secretion

**Figure 3:**
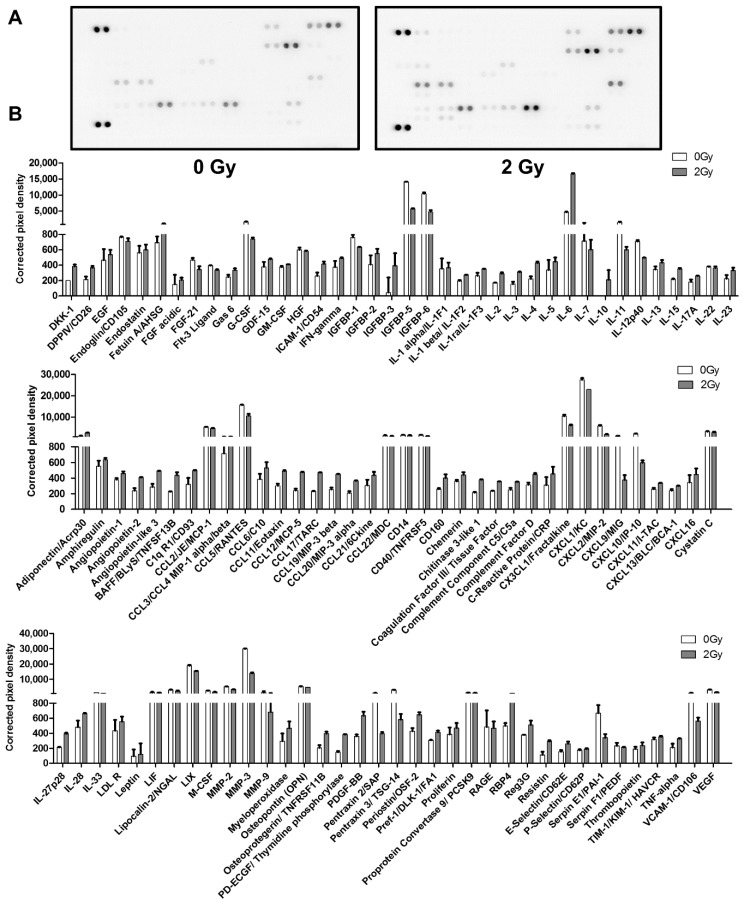
Radiation-induced senescent MLO-Y4 cells secrete multiple SASP components. (**A**) Mouse cytokine microarray membrane analysis was performed to detect the composition of osteocyte SASP in the culture medium of MLO-Y4 cells post irradiation. (**B**) Quantified expression levels of the 111 detected cytokines, expressed as corrected pixel density (*n* = 2). Results are expressed as mean ± SD.

Senescent cells lost their ability to divide and experienced growth arrest, but they were still metabolically active and could increase protein production; this is known as the SASP, which plays an essential role in the secretory regulation of the microenvironment of senescent cells and other surrounding cells. In this study, a mouse cytokine array panel was utilized to examine 111 factors in the supernatant of irradiated MLO-Y4 cells. It was shown that in the supernatant of radiation-induced senescent osteocytes, 72 secretion cytokines increased (Figure 3A,B). These could be broadly classified into the following categories: interleukins (IL-1β, IL-1α, and IL-6), chemokines (CCL6, CCL11, CXCL13, and CXCL16), growth factors (angiopoietin-1, PDGF-BB, EGF, and IGFBP-2), adipokines (leptin, adiponectin, and resistin), and extracellular matrix proteins (DPPIV/CD26, TIM-1, and P-selectin/CD62P); see Table 1.

### 2.4. Radiation-Induced Senescent Osteocytes Perturb BMSCs via a Paracrine Pathway

**Figure 4:**
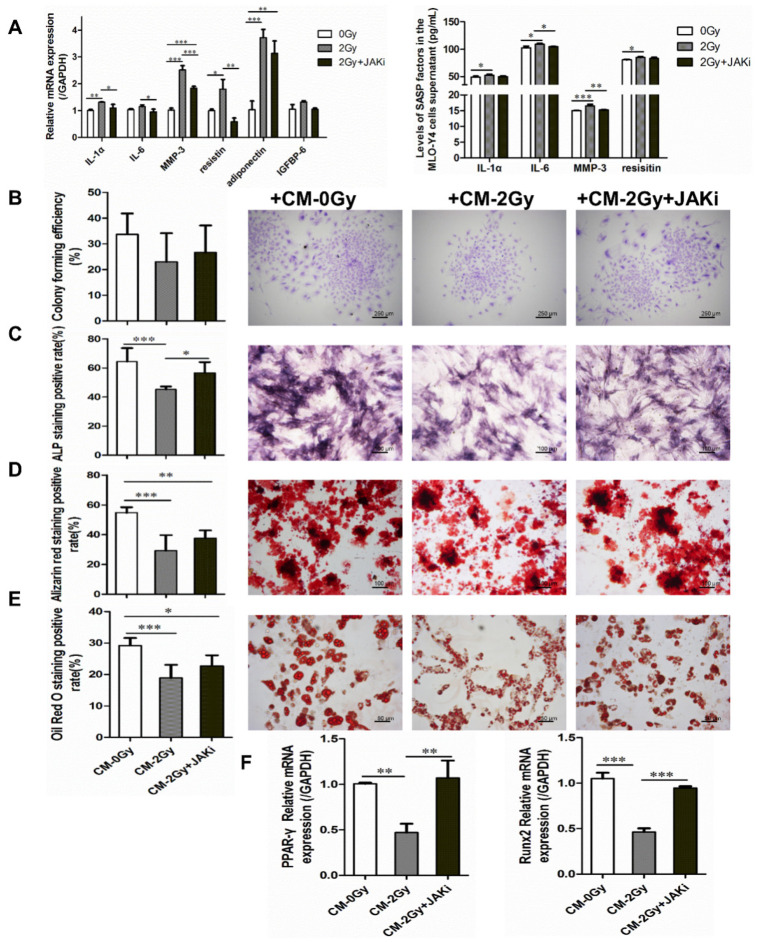
Illustration of the adverse effects of radiation-induced senescent osteocytes on BMSC differentiation via a paracrine pathway. (**A**) JAK1 inhibitor blunting the SASP secretion of radiation-induced senescent MLO-Y4 cells; *n* = 3. (**B**) Colony-forming capacity was influenced after co-culture with CM- 2 Gy, as observed via crystalline violet staining and colony formation rate calculating. Images were acquired in three randomly obtained regions from three independent experiments; magnification = ×40, *n* = 3. (**C**) Representative images and quantitative analysis of ALP-positive expression; magnification = ×100. (**D**) Representative images and quantitative analysis of mineralized nodule area; magnification = ×100, *n* = 6. (**E**) Representative images and quantitative analysis of lipid droplet formation; magnification = ×200, *n* = 6. (**F**) The expression level of osteogenic differentiation-related gene Runx2 and adipogenic differentiation-related gene PPAR-γ in BMSCs co-cultured with CM- 2 Gy or CM- 2 Gy and JAKi. *n* = 3. Results are expressed as mean ± SD (* *p* < 0.05; ** *p* < 0.01; *** *p* < 0.001 vs. 0 Gy).

It was verified that in the irradiated MLO-Y4 cell, the expression of interleukin-1α (IL-1α), interleukin-6 (IL-6), MMP-3, resistin, adiponectin, and IGFBP-6 was significantly increased, and ELISA results also confirm the elevated level of several SASP factors, such as IL-6, IL-1α, MMP-3, and resistin in the irradiated MLO-Y4 cell supernatant. However, when 0.8 μM of JAK1 inhibitor was added during irradiation exposure, the expression levels of SASP factors were significantly reduced, with the most marked decreases in IL-1α, MMP-3, and resistin (* *p* < 0.05; ** *p*  <  0.01; *** *p*  <  0.001; Figure 4A).

Furthermore, the influence of irradiated osteocytes on the colony-formation ability and multidirectional differentiation potential of BMSCs was investigated. The capacity of cell colony formation was mildly disrupted under CM- 2 Gy co-culture. Meanwhile, it was also shown that the potential for both the osteogenic and adipogenic differentiation of BMSCs co-cultured with CM- 2 Gy was significantly decreased. Compared to the control group, the areas of positive alkaline phosphatase, mineralized nodules stained with Alizarin Red S, and lipid droplets stained with Oil Red O were significantly reduced. More interesting is that when 0.8 μM JAK1 inhibitor was added to the irradiated MLO-Y4 cells in order to block SASP secretion, the BMSC differentiation potential inhibition was alleviated to some extent (* *p* < 0.05; ** *p* < 0.01; *** *p* < 0.001; Figure 4B–E). Simultaneously, it was shown that osteogenic and adipogenic differentiation-related genes Runx2 and PPAR-γ were significantly reversed in BMSCs cultured in the CM- 2 Gy+JAKi, which showed a consistent downward trend (** *p*  <  0.01; *** *p*  <  0.001; Figure 4F). The above results verify that senescent osteocytes can perturb BMSCs via a paracrine pathway of SASP.

### 2.5. Bone Aging in Radiation-Induced Bone Damage Mice

**Figure 5:**
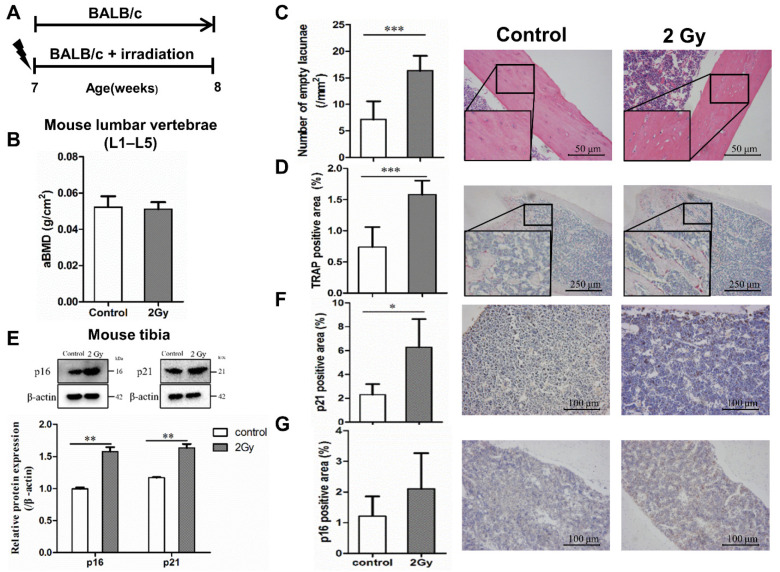
Radiation-induced bone tissue damage and aging. (**A**) Experimental design to test radiation-induced bone tissue senescence in mice. Effect of whole-body 2 Gy irradiation on aging-related bone loss. Seven week-old male BALB/c mice were randomized to receive a single 2 Gy irradiation (*n* = 10) or no irradiation (*n* = 10). The terminal observation time of the experiment was 7 days post irradiation. (**B**) Effect of single 2 Gy irradiation on the areal bone mineral density (aBMD) of the lumbar spine in mice; *n* = 8. (**C**) Effect of single 2 Gy irradiation on osteocyte lacunae in mice after 7 days; magnification = ×200, *n* = 6. (**D**) Effect of single 2 Gy irradiation on TRAP expression in mouse bone tissue after 7 days; magnification = ×40, *n* = 6. (**E**) The expression of senescence-related genes and proteins p21 and p16 in bone tissue was examined by Western blot analysis; *n* = 3. (**F**,**G**) The expression of senescence-related proteins p21 and p16 in mouse bone tissue 7 days post irradiation; magnification = ×100, *n* = 5. Results are expressed as mean ± SD (* *p* < 0.05; ** *p* < 0.01; *** *p* < 0.001 vs. control).

Dual-energy X-ray bone densitometry was used to determine the apparent bone mineral density (aBMD) of the lumbar vertebrae (L1–L5) in the irradiated and control mice. The value of aBMD in the irradiated mice was slightly lower than that in the control mice (0.0503 ± 0.001 vs. 0.0523 ± 0.002 g/cm^2^, *p* > 0.05; Figure 5B). Meanwhile, the lacunae in bone tissue were observed by HE staining, which revealed that the number of empty bone lacunae in the irradiation group was significantly increased (*** *p* < 0.001; Figure 5C). In addition, TRAP staining was used to reflect the bone resorption trend; the results indicate a considerable increase in the TRAP-positive area in the bone tissue of the irradiated group, which means that osteoclastogenesis appeared to be active (*** *p* < 0.05; Figure 5D). Furthermore, the protein expression levels of senescence-related genes p21 and p16 in tibia were elevated significantly after 7 days of 2 Gy whole-body irradiation (** *p*  <  0.01; Figure 5E). Meanwhile, the results of immunohistochemical testing showed a remarkable increase in the number of cells expressing p21 and p16 in the bone marrow cavity of the irradiated femur compared to the control group (* *p* < 0.05; Figure 5F,G).

### 2.6. Changes in Osteocyte and Bone Formation in Radiation-Induced Bone Damage Mice

**Figure 6:**
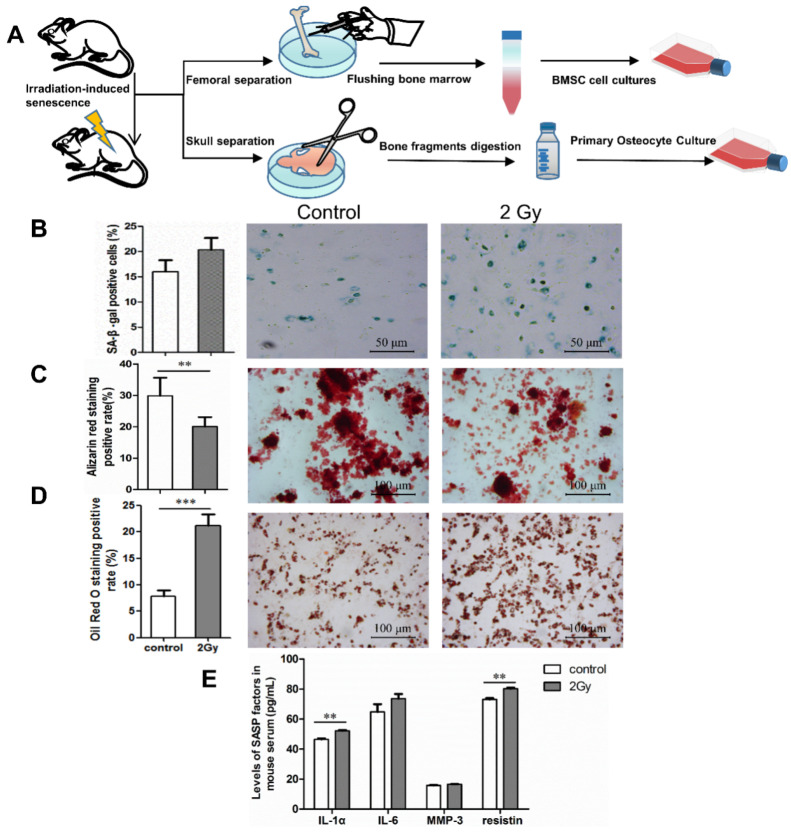
Effect of whole-body irradiation on osteocytes and bone formation. (**A**) Pattern diagram of the experiment. (**B**) Identification of senescence-associated β-galactosidase (SA-β-gal) expression in primary osteocytes extracted from skull bones of mice. Representative images (blue represents SA-β-gal-positive cells) and quantification of SA-β-gal-positive osteocytes from three independent experiments; magnification = ×200, *n* = 3. (**C**,**D**) Effect of whole-body irradiation on bone formation in mice after 7 days. Osteogenic and adipogenic differentiation were induced in vitro by primary BMSCs from mice. Representative images and quantitative analysis of mineralized nodule area and lipid droplet formation are shown; magnification = ×100, *n* = 6. (**E**) Changes in SASP components in mice serum after 7 days post irradiation; *n* = 3. Results are expressed as mean ± SD (** *p* < 0.01; *** *p* < 0.001 vs. control).

The pattern diagram briefly illustrates the femur (for BMSC isolation) and skull bones (for primary osteocyte isolation) isolated from mice (Figure 6A). Following this, bone marrow washed out of the femur was centrifuged and cultured to obtain BMSCs. Meanwhile, the skull bones were digested and cultured to obtain primary osteocytes (Figure 6A). To detect senescence markers in bone tissue, SA-β-gal-positively expressed cells in osteocytes were extracted from mouse skull bones (Figure 6B). To detect levels of SASP in mouse serum, we screened for IL-1α, IL-6, MMP3, and resistin via mouse cytokine array membrane analysis. ELISA results show that at 7 days after irradiation, the levels of IL-1α, IL-6, MMP3, and resistin in the serum of irradiated mice tended to increase compared to the control group (** *p* < 0.01; Figure 6E). The effects of radiation on bone formation were examined using extracted BMSCs to induce mineralized nodules and adipogenic differentiation in vitro. It was found that the in vitro mineralized nodule-formation ability of BMSCs was significantly decreased and the adipogenic-formation ability was significantly increased in the irradiated group (** *p* < 0.01; *** *p* < 0.001; Figure 6C,D).

## 3. Discussion

Recently, cellular senescence and its SASP factors have come to be widely regarded as promising therapeutic targets for aging-related tissue dysfunction, including osteoporosis. With chronological aging, a variety of cells in the bone microenvironment, such as B cells, T cells, myeloid cells, osteoblast progenitors (OP), osteoblasts (OB), and osteocytes (OCY), experience senescence. In this regard, BMSCs and osteocytes are long-living cells that are more susceptible to stress factors that may affect cellular lifespan and lead to cellular senescence [20,21]. In addition, osteocytes are the most abundant cells among a variety of bone microenvironment cells and play critical roles in orchestrating bone remodeling. Therefore, the premature aging of long-living osteocytes induced by stress seems to have a deleterious effect on bone remodeling [20,22,23,24]. In the present study, osteocyte-like MLO-Y4 cells represented by in-vitro-cultured osteocytes exhibited a senescent phenotype accompanying DNA damage under γ-ray irradiation; more crucially, radiation-induced senescent osteocyte-like MLO-Y4 cells could secrete multiple SASP factors that could subsequently alter the osteogenic and adipogenic differentiation potential of BMSCs.

Cellular senescence is a cell fate involving irreversible cell division arrest, tumor suppressor activation, chromatin changes, apoptosis resistance, and protein synthesis increase. It was first proposed by Hayflick and was based on the replicative senescence that occurs on successive cell passages during cell culture. After further research, cellular senescence was divided into telomere replicative senescence and DNA damage-induced senescence [25,26]; the latter mainly comprises senescence caused by ionizing radiation, oxidative stress, and oncogenic gene activation [27,28,29].

The DNA damage response (DDR) mechanism is a common response to the above two types of senescence by activating the p53–p21 and p16–pRB pathways, thus promoting growth arrest [30]. Osteocytes, as the most abundant and longest-living cells in bones, are more prone to accumulate molecular damage with age. Consequently, in the elderly, osteocyte lacunae in cortical bones are accompanied by the degeneration of osteocyte tubules and dendritic connections [17,31]. In our results, it was shown that radiation not only impaired the biological function of osteocyte-like MLO-Y4 cells but also induced cellular senescence and multiple SASP secretions simultaneously. Specifically, cell activity and morphology were damaged after irradiation; exposure to 2 Gy γ-rays significantly damaged the stellate structure of osteocyte-like MLO-Y4 cells and inhibited the elongation of dendrites. Consistent with the changes in dendritic protrusions, the mRNA expression of E11 was downregulated in irradiated MLO-Y4 cells. Meanwhile, irradiation attenuated cell activity and apoptosis in some MLO-Y4 cells, which suggested that MLO-Y4 dysfunction by irradiation could be a critical cause of bone damage [18,32]. Radiation can also cause DNA double-strand breaks (DSBs), which denote the incomplete repair of DNA damage that leads to the persistent DDR and permanent cell growth arrest that is cellular senescence [33]. In addition, after the induction of γ-H2AX foci, an important protein marker for the detection of DSB sites [34] and the generation of SAHF by 2 Gy of γ-radiation was detected, the expression of senescence-associated genes p21 and p16 was upregulated in MLO-Y4 cells post irradiation. Thus, our outcomes highlight that in addition to causing cellular senescence, irradiation could impair the morphology and viability of osteocyte-like MLO-Y4 cells.

During their accumulation, senescent cells may secrete a series of factors called the SASP, which can induce inflammation, activate the immune system, and promote the spread of senescence to other cells, both locally and systemically, by paracrine or endocrine means [35]. Though senescent osteocytes appear to be the main sources of the SASP in bone tissues and the significantly higher expression of multiple SASP markers in ageing bone loss [20,36], the expression pattern and hallmark of the SASP in stress-induced senescence remain unclear, thus leaving a large gap in the knowledge for a desirable intervention targeting the cellular senescence. Therefore, to characterize the composition and function of the osteocyte SASP in stress-induced senescence, we analyzed cytokine expression in a cell culture medium using an antibody microarray as a first screening. The results reveal that the osteocyte SASP, comprising multiple characteristic factors such as pro-inflammatory cytokines (IL-6 and IL-1α), metalloproteinase (MMP-3), adipokines (resistin and adiponectin), and insulin-like growth factor (IGFBP-6), was markedly upregulated in irradiated osteocyte-like MLO-Y4 cells. As noted earlier, one of the most prominent cytokines in SASP is IL-6, which is a pleiotropic pro-inflammatory cytokine. It has been reported that IL-6 is correlated with DNA damage and oncogenic stress-induced senescence in mouse and human keratinocytes, fibroblasts, and melanocytes [37,38,39]. Additionally, it appears that IL-6 secretion is under the direct control of persistent DNA damage signals (ATM and Chk2) and senescent cells could directly affect neighboring normal cells through IL-6 expression [40]. Another important cytokine, IL-1α, can affect neighboring cells through cell surface receptors that mainly serve to activate the nuclear factor κB and activate protein 1 pathways [40]. It was reported that chemokines highly expressed in aging bone tissue are comprised of IL-8 (CXCL15), MCP-1 (CCL2), and RANTES (CCL5) [36]. The IGF/IGF receptor network may also be engaged in the contribution of senescent cells to their microenvironment. It was documented that IGFBP-5 seems to play a critical role in the control of cellular senescence and inflammation [41]. In addition, it was proposed that adipokines (resistin and leptin) could be used as markers of biological aging [42]. These results allow us to hypothesize that senescent osteocytes and their SASPs may contribute to radiation-induced bone loss.

Given the critical role of dysfunctional senescent osteocytes and their SASPs in regulating bone remodeling, the targeted elimination of SASP secretion may potentially reverse the degenerative differentiation of BMSCs, enhance bone formation, and consequently prevent radiation-induced bone loss. The JAK pathway is critical in the production of cytokines, while JAK1 and JAK2 primarily regulate inflammatory signaling. In the present study, we evaluated the role of the JAK1 inhibitor, which can restrain SASP secretion from senescent cells by inhibiting the JAK pathway. The results show that the JAK1 inhibitor could block the expression of SASP factors in a culture medium of radiation-induced senescent MLO-Y4 cells and downregulate IL-1α, MMP-3, resistin, and adiponectin. In addition, it was observed that normal BMSCs co-cultured with CM- 2 Gy containing multiple SASP components exhibited diminished colony-formation abilities and impaired differentiation potential toward osteogenesis or adipogenesis. Additionally, CM- 2 Gy and JAKi action was noted to be able to alleviate the deleterious effect of radiation-induced senescent MLO-Y4 cells and SASP on BMSCs. These results suggest that the accumulation of SASPs by radiation-induced senescent osteocytes might play a negative role in the osteogenic and adipogenic differentiation of BMSCs.

Cellular senescence has emerged as a fundamental senescent mechanism for the prevention or mitigation of age-related pathologies, including osteoporosis. In a variety of model animals, it has been demonstrated that the selective removal of senescent cells or interference with their SASP secretions could be an effective strategy in preventing or treating age-related diseases. Several common senolytic drugs, including dasatinib plus quercetin, ABT-737, and BET inhibitors, could ameliorate the symptoms of age-related degenerative pathologies by reducing the secretion of SASP and selectively inducing senescent cell apoptosis [43]. Thus, it is important to provide an effective animal model of cellular senescence and tissue dysfunction for preclinical research.

Animal models of aging could play an essential role in the research of aging mechanisms, as well as in clinical studies of anti-aging therapy. Current widely used aging animal models include the spontaneous aging model (natural aging model and senescence accelerated mouse [44]) and the induced aging model (irradiation-induced aging model [45], β-amyloid-induced aging model [46], transgenic aging model [47], and D-galactose-induced accelerated aging model [48]). In our study, the whole-body irradiation of 2 Gy γ-rays was used as a stressor to successfully induce cellular senescence and bone loss. The results revealed that along with the increase in empty lacunae and the enhancement of osteoclastogenesis activity, senescence was confirmed by cytochemical staining and Western blot analysis for classical senescence markers of p16 and p21, and it was further validated by an increase in the expression of senescence-associated-β-galactosidase activity in primary osteocytes from the skull bones of radiation-induced bone loss mice. Additionally, a decrease in the osteogenic capacity but increase in the adipogenic capacity of BMSCs in irradiated mice was observed. In conclusion, in vivo experiments demonstrated that senescent osteocytes and their secreted SASPs might comprise an important mechanism causing the degenerative differentiation of BMSCs. The authors of this study also successfully developed a mice model of stress-induced senescence. However, aging is a very complex biological process, so it is necessary to make optimal use of different animal models to study aging and preferably clinically translate the research results to provide healthy aging for human beings.

Ultimately, our findings demonstrate that in addition to causing cellular senescence, irradiation can impair the morphology and viability of osteocyte-like MLO-Y4 cells. In addition, radiation-induced senescent osteocytes can secrete multiple characteristic SASP factors that can subsequently perturb BMSCs’ differential potential via the paracrine signaling of SASP, which was also demonstrated by in vivo experiments. These results allow us to hypothesize that senescent osteocytes and their SASPs may contribute to radiation-induced bone loss.

## 4. Materials and Methods

### 4.1. Cell Culture and Irradiation

Mouse osteocyte-like MLO-Y4 cells were obtained from Cell Bank (Shanghai Institutes for Biological Sciences, Chinese Academy of Sciences, Shanghai, China). The cell lines were used to study the properties of osteocytes and their biological function in vitro [49]. The osteocyte-like MLO-Y4 cells were inoculated and cultured on plates coated with rat tail collagen type I (Solarbio, Beijing, China) in α-minimum essential medium (α-MEM; Gibco, Eggenstein, Germany) containing 5% fetal bovine serum (FBS; Gibco), 5% calf serum, and 1% penicillin/streptomycin (Sigma-Aldrich, St. Louis, MO, USA) in a humidified incubator at 37 °C with 5% CO_2_ [32]. Prior to irradiation, cells were incubated in plates overnight, and cells that adhered to the wall were irradiated with 2 Gy using ^137^Cs γ-rays (Nordyon, Ottawa, ON, Canada). Unirradiated (0 Gy) cells were positioned in the irradiator for the same duration without irradiation. After irradiation, all cells were renewed with a fresh culture medium, continued to be cultured for three days, and were then inoculated for subsequent experiments.

### 4.2. Radiation-Induced Morphological and Functional Changes in MLO-Y4

Phalloidin staining for dendritic morphology: Radiation-induced changes in osteocyte morphology were assayed by phalloidin staining. F-actin was stained with tetramethylrhodamine (TRITC)-phalloidin (Solarbio, Beijing, China), and nuclei were stained with 4′,6-diamino-2-phenylindole (DAPI; Dojindo, Kumamoto, Japan). Images were taken with a Leica fluorescence microscope (Leica Microsystems, Wetzlar, Germany). Simple PCI software (C-imaging, Lake Oswego, OR, USA) was used to evaluate the lengths of the dendritic protrusions of MLO-Y4 cells.

CCK8 assay for cell viability: MLO-Y4 cells were seeded at a density of 3000 cells/well on 96-well plates and irradiated after 24 h of culture, followed by culture for 3 days. Then, the effect of γ-rays on cell viability was measured using a Cell Counting Kit-8 assay (C0037, Beyotime Biotechnology, Shanghai, China). Briefly, the culture solution was discarded, the prepared CCK8 reaction working solution was added to each well, and the cells were incubated for 2 h at 37 °C in the dark. The absorbance of the colorimetric dye was measured at 450 nm by a microplate reader (Bioteck, Vicenza, Italy).

Flow cytometry assay for apoptosis and necrosis: Cell flow cytometry assays were used to detect apoptosis vs. necrosis in irradiated MLO-Y4 cells via the fluorescent labeling of markers on cell surfaces. Briefly, MLO-Y4 cells were inoculated in 6-well plates and treated with irradiation (0 and 2 Gy), followed by culture for 3 days. Cells were harvested and then gently suspended in a mixture of 40 μL of binding buffer, 5 μL of fluorescein thiocyanate labeled by Annexin V-FITC, and 5 μL of propidium iodide (PI). After incubation for 15 min at room temperature in the dark, the supernatant was discarded after centrifugation and cells were resuspended with 50 μL of binding buffer per well. Finally, cells were tested by flow cytometry using Cellometer^®^ K2 (Nexcelom, Lawrence, MA, USA) and then analyzed for apoptosis and necrosis fraction by employing De Novo FCS Express software.

### 4.3. Radiation-Induced Cellular Senescence and Its Secretory Phenotype

Detecting aging-related genes: The expression of the aging-related genes p16 and p21 in irradiated osteocytes was detected with Western blot and RT-*q* PCR. The detailed procedure is described below.

DAPI staining for detecting SAHF formation: SAHF, a marker of chromatin structure damage, was used as one of the critical markers for identifying senescent cells. The cells were plated on cover slips and irradiated, and then they were cultured for 3 days. Cells were rinsed three times with PBS and fixed with 4% paraformaldehyde for 10 min at room temperature, and then they were washed three times with PBS and incubated with DAPI for 1 min in the dark. Cell nuclei were analyzed with a Leica TCS SP8 confocal microscope (Leica Microsystems, Wetzlar, Germany).

Immunofluorescence detection for γ-H2AX foci: We used an immunofluorescence assay to detect γ-H2AX foci, a marker of DNA damage. The γH2AX foci were tested according to the previous description [50], and then the γ-H2AX foci number was quantified with a Leica fluorescence microscope (Leica Microsystems, Wetzlar, Germany).

Detection and analysis of multiple cytokines: A Mouse XL Cytokine Array Kit (R&D; Cat ARY028, U.S.) was used to simultaneously detect the levels of 111 different cytokines in 500 μL of culture supernatant [51]. Among these 111 factors were cytokines, chemokines, and growth factors. The signals were detected with a chemiluminescence imager (Chemi Scope 6300), followed by quantitative analysis with the HLImage++ computer vision system (1997, Western Vision Software, Salt Lake City, UT, USA). The relative expression levels of multiple cytokines in a cell culture medium were determined by the following process. First, the membranes were prepared and incubated with the prepared samples, followed by the incubation of the membranes with a Detection Antibody Cocktail; then, the membrane array was incubated with streptavidin–HRP, the membrane array was further developed utilizing chemical reagents 1 and 2, and, finally, the array film was placed in an autoradiographic film cassette and exposed to X-ray film for imaging.

Enzyme-linked immunosorbent assay for SASP: An ELISA kit (Enzyme-Linked Biotechnology, Shanghai, China) was used to detect the major components of SASP in the culture supernatant of radiation-induced senescent MLO-Y4 cells and mouse serum, such as IL-6, IL-1α, MMP-3, and resistin. Briefly, irradiated MLO-Y4 was incubated under normal conditions for 3 days and then incubated in serum-free medium for an extra 24 h. The supernatant was then collected for an enzyme-linked immunosorbent assay according to the manufacturer’s protocol.

### 4.4. The Effect of Radiation-Induced Senescent Osteocytes on BMSCs

Collection of conditioned medium (CM) with irradiated MLO-Y4: Unirradiated and 2 Gy-irradiated MLO-Y4 were cultured for 3 days and were then continually incubated in a serum-free medium for 24 h. Culture supernatants from sham MLO-Y4 and irradiated MLO-Y4 were extracted as CM- 0 Gy and CM- 2 Gy, respectively. In the case of JAK1 inhibitor (JAKi) treatment, irradiated MLO-Y4 cells were treated with 0.8 μm of JAK1 inhibitor for 72 h, washed three times with PBS, and then cultured in a serum-free medium for 24 h. Supernatants were extracted as CM- 2 Gy+JAKi.

Isolation and culture of BMSCs: Primary BMSCs were isolated from 7-week-old male BALB/c mice (weight: ~23 g) purchased from the Department of Experimental Animals at Fudan University (Shanghai, China). Euthanasia was conducted through the intraperitoneal injection of anesthetic, followed by the separation of femur and tibia. Then, total bone marrow cells were obtained according to the previously described method [52]. The sediment cell was resuspended with α-MEM and then inoculated in T25 cell flasks. After incubation in a humidified incubator at 37 °C with 5% CO_2_ for 48 h, the adherent cells were gathered as BMSCs.

BMSC colony-forming unit (CFU) formation: The passage 3 BMSCs were seeded into 6 cm Petri dishes at a density of 10^3^ cells/dish and slightly shaken in the cross direction to ensure that the cells spread evenly. After 24 h of affixation to the wall, the α-MEM (GIBCO) containing 50% CM (0 Gy, 2 Gy, and 2 Gy+JAKi) was replaced and then cultured in a humidified incubator at 37 °C with 5% CO_2_. Two weeks later, cell colonies were visible, fixed with methanol for 15 min, and stained with crystal violet (C8470; Solarbio) for 10 min; then, CFU was determined.

Osteogenic differentiation potential for BMSCs: The passage 3 BMSCs in well-grown state were collected and inoculated in osteogenic induction medium (α-MEM comprising 15% FBS, 1% penicillin–streptomycin, 50 mg/L of ascorbic acid, 0.01 μM of dexamethasone (ST1254, Beyotime Biotechnology), and 10 mM of β-glycerophosphate (Sigma-Aldrich)) containing 50% CM (0 Gy, 2 Gy, and 2 Gy+JAKi). The medium was freshly exchanged every 2 days. On the 7th day of osteogenic induction culture, cells were fixed with 2.5% glutaraldehyde (Sigma-Aldrich) for 15 min and washed three times with PBS. According to the protocol of the BCIP/NBT Alkaline Phosphatase Color Development Kit (C3206; Beyotime Biotechnology), the staining is described in detail in the previous description [53]. Simultaneously, ALP activity was examined using the Alkaline Phosphatase Assay Kit (P0321, Beyotime Biotechnology) according to the manufacturer’s instructions. The ALP activity was calculated with a p-nitrophenol standard curve by measuring the absorbance at 405 nm. In addition, to evaluate the differential ability of functional osteoblasts, the in vitro formation of mineralized nodules was measured. The passage 3 BMSCs were inoculated in 48-well plates with a cell density of 3 × 10^4^ cells per well. After 21 days of osteogenic induction, mineralized nodules were counted by Alizarin Red staining. Briefly, cells were fixed in 95% ethanol for 10 min, and then a 0.2% Alizarin Red S (Sigma-Aldrich) solution (pH = 8.3) was added and incubated for 1 h at 37 °C. Cells were flushed with PBS and dried by shaking. The area of mineralization nodules was measured under a microscope.

Adipogenic differentiation potential for BMSCs: The passage 3 BMSCs were inoculated in 48-well plates with a cell density of 3 × 10^4^ cells per well, and then they were inoculated in adipogenic induction medium (α-MEM containing 10% FBS, 1% penicillin–streptomycin, 0.1 mM of 3-isobutyl-1-methylxanthine (ST1398, Beyotime Biotechnology), 0.1 mM of indomethacin (YZ-100258; Solarbio), 1 μM of dexamethasone, and 10mg/L of insulin (P3375, Beyotime Biotechnology)) containing 50% CM (0 Gy, 2 Gy, and 2 Gy+JAKi). Oil Red O (Sigma-Aldrich) staining was used to observe the positive cells after 16 d of adipogenesis induction. Briefly, cells were fixed with 4% paraformaldehyde (P1110; Solarbio), gently rinsed with 60% isopropanol, stained with Oil Red O for 30 min at room temperature, and washed with distilled water. The area of Oil Red O staining was measured under a microscope.

### 4.5. Osteocyte Senescence in a Mouse Model of Radiation-Induced Bone Loss

Animals and irradiation protocol: BALB/c mice were randomly divided into control and irradiated groups, with 10 mice in each group. The mice were placed in a special circular irradiation container, and their whole bodies were exposed to X-rays for single irradiation of 2 Gy, while the control group was placed on the irradiation platform with no irradiation treatment. Irradiation requirements: tube voltage of 220 kV, tube current of 12.0 mA, source surface distance of 50.0 cm, and dose rate of 121.3 cGy/min. Mice were euthanized 7 days post irradiation, and then serum was collected and tissues were immediately dissected for further phenotyping. During the experiment, the rearing environment was a 12 h light/dark cycle with constant temperature (20–25 °C) and free diet and water.

Lumbar spine bone mineral density: Dual-energy X-ray absorptiometry (QDR 4000, Hologic, Bedford, MA, USA) was used for the determination of bone mineral density in the L1–L5 area of the lumbar spine.

Histomorphometric analysis of bone tissue: Briefly, mouse femurs were isolated, fixed in 4% paraformaldehyde for 24 h, decalcified with 10% EDTA, paraffin embedded, and cut into 4 μm-thick sections. All bone sections were stained using standard HE staining and TRAP staining techniques. In each group, six sections of femoral specimens were selected for observation and analyzed with Simple PCI software.

Immunohistochemical staining for bone tissue: Bone tissue sections were dewaxed and then heated in citrate antigen retrieval solution (P0081, Beyotime Biotechnology) at 100 °C for 20 min, followed by 3% BSA for 30 min. Following the manufacturer’s instructions, the appropriate amount of primary antibody (p21 and p16) was added and incubated at room temperature for 2 h. The sections were then coated with polymeric anti-rabbit poly-HRP-IgG for 8 min. Finally, DAB staining with hematoxylin re-staining was performed. The tissues were then dehydrated in ethanol, followed by xylene, and then neutral resin blocking was performed to observe the expression of target proteins in bone tissue under microscope.

Isolation of primary osteocytes for culture: Osteocytes were isolated from the calvarium of BALB/c male mice 7 days post irradiation. The mouse cranium was aseptically separated, the soft tissue surrounding the bone was removed, and the periosteum was scraped off with a sterile scalpel. The bones were cut into fragments after being repeatedly removed from wells in sterile phosphate-buffered saline (PBS) containing 1% penicillin/streptomycin (Sigma-Aldrich, St. Louis, MO, USA). These fragments were sequentially digested and the supernatant was collected. Mixed supernatants were centrifuged and resuspended in α-MEM medium containing 5% fetal bovine serum, 5% calf serum, and 1% penicillin/streptomycin, which were inoculated together with the final bone fragments in dishes coated with rat tail collagen type I (Solarbio, Beijing, China) and incubated in a humidified incubator at 37 °C and 5% CO_2_. A more detailed protocol for primary osteoblast extraction can be found in our previous publication [50]. The production of dendritic osteocytes was microscopically visible after 3–7 days. Cells from passages 3–5 were used in the experiments.

Senescence-associated β-galactosidase staining for primary osteocytes: Cells isolated from irradiated mice were placed on 6-well plates with 1 × 10^5^ cells per well and incubated at 37 °C for 24 h. The senescent osteocytes were assessed using the SA-β-gal staining kit (C0602; Beyotime Biotechnology) according to the manufacturer’s instructions [54]. SA-β-gal-positive cells were assessed by counting 200 cells from randomly taken images, and the results are expressed as percentages.

### 4.6. RNA Extraction and Real-Time Quantitative PCR

Cells were cultured in six-well plates, total RNA was extracted using the Simply P Total RNA Extraction Kit (BSC52S1; Bioflux, Beijing, China), and cDNA was synthesized using FastKing gDNA Dispelling RT SuperMix (KR118-02; Tiangen Biotech, Beijing, China) [52]. Real-time PCR was performed using the ABI QuantStudio 5 (Applied Biosystems, Carlsbad, CA, USA) with PowerUp SYBR Green Master Mix (Invitrogen; Thermo Fisher Scientific, Inc., Waltham, MA, USA) in a 10 μL reaction system according to the manufacturer’s protocol. The primer sequences of the target genes are indicated in Table 2.

### 4.7. Western Blot Analysis

Cells were lysed using a RIPA lysis buffer (Beyotime Institute of Biotechnology, Shanghai, China) containing protease inhibitors. The tibiae were placed into a freeze grinder for grinding and then added to a lysis buffer and placed on ice for 30 min. After lysates were harvested by centrifugation at 12,000× *g* for 10 min at 4 °C, total cellular protein concentrations were determined using the BCA Protein Assay Kit (Beyotime Institute of Biotechnology, China) according to the manufacturer’s instructions. Protein (20–30 μg) was subjected to 12.5% SDS–polyacrylamide gel electrophoresis (PG113; Epizyme Biotech) and transferred onto polyvinylidene difluoride (PVDF) membranes (Millipore, Billerica, MA, USA). Whole membranes were blocked with 5% non-fat milk for 30 min at room temperature, and then immunoblotting was conducted with primary antibodies (1:1000) as follows: E11 (ab256564), p21 (ab109199), p16 (ab51243), and β-actin (AF7018). After incubation overnight at 4 °C, the membranes were incubated with anti-rabbit and anti-mouse antibodies (A0208, A0216; 1:2000) and developed with an ECL Kit (D3308; Beyotime Institute of Biotechnology, China). The quantitative analysis was carried out with Image J software (National Institutes of Health, Bethesda, MD, USA), and β-actin was used to normalize the expression of protein levels.

### 4.8. Statistical Analysis

The statistical analysis of the data was performed with IBM SPSS Statistics 20 (SPSS Inc., Chicago, IL, USA) and GraphPad Prism 5.0 (GraphPad Software Inc., San Diego, CA, USA). All data are expressed as mean ± standard deviation (SD). To evaluate the statistically significant differences between groups, an independent sample *t*-test or one-way ANOVA was adopted. Following ANOVA, the Tukey’s multiple comparison test was used to adjust for multiple comparisons. * *p* < 0.05 was considered statistically significant.

## Figures and Tables

**Table 1:** Changes in SASP pattern in radiation-induced senescent osteocytes.

Category	SASP Factors	Change Pattern
Interleukins (IL)	IL-1β, IL-1α, IL-2, IL-3, IL-4, IL-5, IL-6, IL-10, IL-13, IL-15, IL-17A, IL-23, IL-27p28, IL-28	↑
	IL-7, IL-11, IL-12p40, IL-22, IL-33	↓
Chemokines(CXCL, CCL)	CCL3/CCL4 MIP-1 alpha/beta, CL6/C10, CCL11/eotaxin, CCL12/MCP-5, CCL17/TARC, CCL19/MIP-3 beta, CCL20/MIP-3 alpha, CCL21/6Ckine, CXCL11/I-TAC, CXCL13/BLC/BCA-1, CXCL16	↑
	CCL5/RANTES, CCL2/JE/MCP-1, CCL22/MDC, CX3CL1/fractalkine, CXCL1/KC, CXCL2/MIP-2, CXCL9/MIG, CXCL10/IP-10, LIX	↓
Other inflammatory factor	GDF-15, GM-CSF, IFN-gamma, BAFF/BLyS/TNFSF13B, TNF-alpha, coagulation factor III/tissue factor	↑
	G-CSF	↓
Growth factors and regulators	Amphiregulin, angiopoietin-1, angiopoietin-2, angiopoietin-like 3, EGF, fetuin A/AHSG, FGF acidic, PD-ECGF/thymidine phosphorylase, PDGF-BB, proliferin, IGFBP-2, IGFBP -3	↑
	HGF, VEGF, osteopontin (OPN), FGF-21, IGFBP-1, IGFBP-5, IGFBP-6, Flt-3 ligand	↓
Proteases and regulators	Myeloperoxidase, complement factor D, RBP4, fetuin A/AHSG	↑
	M-CSF, MMP-2, MMP-3, MMP-9 proprotein convertase 9/PCSK9, serpin F1/PEDF, RAGE, cystatin C, serpin E1/PAI-1	↓
Soluble or shed receptors or ligands	ICAM-1/CD54, osteoprotegerin/TNFRSF11B, chemerin, chitinase 3-like 1, Gas6, LDL R, C-reactive protein/CRP	↑
	LIF	↓
Adipokines	Adiponectin/Acrp30, resistin, leptin, Pref-1/DLK-1/FA1	↑
	—	↓
Extracellular matrix protein	CD160, complement component C5/C5a, TIM-1/KIM-1/HAVCR, periostin/OSF-2, endostatin, thrombopoietin, Reg3G, P-selectin/CD62P, Dkk-1, WISP-1/CCN4, coagulation factor III/tissue factor, C1q R1/CD93, DPPIV/CD26, E-selectin/CD62E	↑
	CD14, CD40/TNFRSF5, endoglin/CD105, pentraxin 2/SAP, pentraxin 3/TSG-14, lipocalin-2/NGAL, VCAM-1/CD106,	↓

**Table 2:** Primer sequences for quantitative reverse-transcription polymerase.

Target Genes	Primer Sequence
E11	S 5′-CTGGCCTGAGGTCATCTTGT-3′ A 5′-TCCATCCCCACCAACAAGTG-3′
p16	S 5′-CGCAGGTTCTTGGTCACTGT-3′ A 5′-TGTTCACGAAAGCCAGAGCG-3′
p21	S 5′-CCTGGTGATGTCCGACCTG-3′ A 5′-CCATGAGCGCATCGCAATC-3′
Adiponectin	S 5′-CCAGGAAGAAACCACCGGA-3′ A 5′-GAAATCAGGAAGGCTGCCAAG-3′
Resistin	S 5′-CATGCCATGGGGTCCAGCATGCCACTGT-3′ A 5′-CCCAAGCTTTCAGGAAGCGACCTGCA-3′
IL-6	S 5′-ATGAACAACGATGATGCACTTG-3′ A 5′-GGTACTCCAGAAGACCAGAGG-3′
IL-1α	S 5′-CTGAAGAAGAGACGGCTGAGT-3′ A 5′-CTGGTAGGTGTAAGGTGCTGAT-3′
MMP-3	S 5′-AGGGATGATGATGCTGGTATG-3′ A 5′-AACACCACACCTGGGCTTAT-3′
IGFBP-6	S 5′-GCAGCAGCTCCAGACTGA-3′ A 5′-CATTGCTTCACATACAGCTCAA-3′
GAPDH	S 5′-AGGTCGGTGTGAACGGATTTG-3′ A 5′-GGGGTCGTTGATGGCAACA-3′
Runx2	S 5′-TGCCACCTCTGACTTCTGC-3′ A 5′-GTCAAGGGTCCGTAAAGTAG-3′
PPAR-γ	S 5′-GGAAGACCACTGCATTCCTT-3′ A 5′-GTAATCAGCAACCATTGGGTCA-3′

## Data Availability

The datasets generated and analyzed in the present study are available from the corresponding author on reasonable request.

## Abbreviations

SASP	Senescence-associated secretory phenotype
MCP	Membrane cofactor protein
EGF	Endothelial growth factor
HGF	Hepatocyte growth factor
FGF	Fibroblast growth factor
VEGF	Vascular endothelial growth factor
IGFBP	Insulin-like growth factor binding protein
MMP	Matrix metalloproteinase
ICAM	Intercellular adhesion molecule
IL	Interleukin
PPAR-γ	Peroxisome proliferator-activated receptor-γ
